# Study on relationships among deep vein thrombosis, homocysteine & related B group vitamins

**DOI:** 10.12669/pjms.312.6049

**Published:** 2015

**Authors:** Meral Ekim, Hasan Ekim, Yunus Keser Yilmaz, Bahadir Kulah, M. Fevzi Polat, A. Yesim Gocmen

**Affiliations:** 1 Meral Ekim, MD, PhD. Bozok University School of Health, Yozgat, Turkey; 2 Hasan Ekim, MD. Department of Cardiovascular Surgery, Bozok University School of Medicine, Yozgat, Turkey; 3 Yunus Keser Yilmaz, MD. Department of Cardiovascular Surgery, Bozok University School of Medicine, Yozgat, Turkey; 4 Bahadir Kulah, MD. Department of Surgery, Bozok University School of Medicine, Yozgat, Turkey; 5 M. Fevzi Polat, PhD. Department of Biochemistry, Bozok University School of Medicine, Yozgat, Turkey; 6 Yesim Gocmen, PhD. Department of Biochemistry, Bozok University School of Medicine, Yozgat, Turkey

**Keywords:** Deep Venous Trombosis, Hyperhomocysteinemia, B Group Vitamin

## Abstract

**Objectives:**

Hyperhomocysteinemia has been considered as a potential risk factor for deep venous thrombosis (DVT) but it is still controversy. We aimed to evaluate the prevalence of hyperhomocysteinemia in patients with DVT. Our second objective was to document the prevalence of folate, Vitamin B6, and Vitamin B12 level in this patient population.

**Methods:**

Sixty patients with DVT aged from 23 to 84 years, were assessed regarding demographic characteristics, serum levels of homocysteine, folate, vitamin B12, and vitamin B6. The diagnosis of DVT was based upon Wells scoring system and serum D-dimer level and confirmed by deep venous Doppler ultrasonography of the lower limbs.

**Results:**

Mean serum homocysteine levels were found significantly higher in patients over the age of 40 years (10.81±4.26 µmol/L vs 9.13±3.23 µmol/L). Of all the patients, 9 patients had homocysteine level above the 15µmol/L, 26 had folic acid level below 3 ng/ml, one had vitamin B12 level below 150 pmol/L, and two had vitamin B6 level below 30 nmol/L. In the hyperhomocysteinemic group, five patients had low folic acid level, one had low vitamin B12 level, and two had low vitamin B6 level.

**Conclusions:**

Hyperhomocysteinemia, in women older than 40 years, may be a risk factor for DVT. Folic acid deficiency may also influence serum homocysteine concentrations. Folate therapy may be offered to the patients with DVT. However further studies are required to clarify the underlying molecular mechanisms.

## INTRODUCTION

Venous thromboembolism (VTE) is the third most common vascular disease after myocardial infarction and ischemic stroke. It is well known that, individual coagulation factors are the best markers of venous thrombotic risk and, in part up to 50% determined by genetic factors. Lifestyle and environmental factors have also effect on thrombotic events.^1^

Hyperhomocysteinemia is a disorder of methionine metabolism and proposed to be a modifiable risk factor of myocardial infarction, peripheral arterial thrombosis as well as deep vein thrombosis and pulmonary embolism.^2^ Most of the reports related to arterial disease associated with mildly increased homocysteine level. On the contrary, there are limited and conflicting publications related to venous system thrombosis associated with homocysteine level.^3^

Among venous thrombosis risk factors, hyperhomocysteinemia has been considered as a potential factor by most studies, but is still under debate.^4^ Nevertheless, high prevalence of hyperhomocysteinemia was demonstrated in venous thrombosis patients in several case-control studies and meta-analyses.^5^ Hyperhomocysteinemia due to reduced B-group vitamins (folic acid, vitamin B12, and vitamin B6) levels is thought to be a part of the pathophysiologic link between B-group vitamins and venous thrombosis.^6^ Besides, Methylenetetrahydrofolate reductase (MTHFR) gene mutations were supposed to increase the risk of DVT. Increased hyperhomocysteinemia (sometimes be related with MTHFR mutations) seems to be responsible for increased risk of thrombosis.^7^ The homozygote polymorphism of MTHFR (C677T) is the most common hereditary defect of the remethylation process.^8^ The coherence of these findings probably supports that hyperhomocysteinemia is clinically significant in venous thrombosis.^5^

The aim of our study was to assess the prevalence of hyperhomocysteinemia in our urban population with DVT. Our second objective was to document the prevalence of folate, Vitamin B6, and Vitamin B12 level in this patient population.

## METHODS

Between May 2013 and February 2014, 60 consecutive patients with deep venous thrombosis admitted to Bozok University Hospital were included in our study. The study was performed in accordance with the regulations of the ethics committee of Bozok University and the declaration of Helsinki. The informed consent was obtained from every participating patient.

As conditions like drug usage, hypothyroidism, pernicious anemia, inflammatory renal disease, respiratory insufficiency, a history of antiphospholipid syndrome, and sepsis may interfere with the blood level of homocysteine levels, those patients were excluded from the study. Patients on folic acid, vitamin B12, and vitamin B6 treatment were also excluded. Medical records of sixty patients with unprovoked DVT were reviewed.

The diagnosis of DVT was based upon Wells scoring system and serum D-dimer level and confirmed by deep venous Doppler ultrasonographic examination. The findings of physical examination, laboratory findings, electrocardiography and chest radiograph were all recorded.

All patients were asked to refrain from smoking and from taking vigorous exercise prior to blood sampling. The total homocysteine, vitamin B12, vitamin B6, and folic acid levels were measured on heparinized blood samples, collected after a 12-hours fasting. The blood samples placed on ice and were centrifuged within one hour after a traumatic venipuncture. Serum samples were frozen at -70º C until analyses. For biochemical analyses, homocysteine, folic acid, vitamin B12, and vitamin B6 levels were measured by EIA method (USCNlife-EİAab, China). The absorbance values obtained from the device were substituted into the calibration chart to prepare the results of samples. Additionally, D-dimer (cut off value 500 ng/ml) was assayed in routine biochemistry laboratory.

The homocysteinemia levels above 15 µmol/L were considered as hyperhomocysteinemia. Values between 15 and 30 µmol/L were considered as mild, values between 30 and 100 µmol/L were regarded as intermediate, and values above 100 µmol/L were regarded as severe hyperhomocysteinemia, as previously described.^9^

Blood concentrations of folic acid less than 3ng/ml, vitamin B_12_ less than 150 pmol/L, and vitamin B6 less than 30 nmol/L are accepted as low vitamin level. The results were expressed as the mean ± standard deviation. The Student t test was used for statistical analysis. A p value of less than or equal to 0.05 was accepted as being statistically significant.

## RESULTS

During study period, 60 patients with DVT met the inclusion criteria and were enrolled in the study. There were 31 male and 29 female patients ranging in age from 23 to 84 years, with a mean age of 50.5 years old. After diagnosis, all patients were treated LMWH and warfarin sodium (Coumadin). Increased D-dimer levels were noted in 48 (80%) patients. All patients had normal liver and renal functions.

Of the 60 patients, 9 patients had hyperhomocysteinemia, 26 had low folic acid level, one had vitamin low B12 level, and two had low vitamin B6 level. Mean Homocysteine levels were 9.86±6.22 µmol/L in females and 8.85±5.24 µmol/L in males. The difference was insignificant (p>0.05). But, mean homocysteine concentration was significantly higher in patients over the age of 40 years (10.81±4.26 µmol/L) than younger patients (9.13±3.23 µmol/L) (p<0.05). Mild hyperhomocysteinemia were detected in eight patients whereas intermediate hyperhomocysteinemia was noted only in one patient.

The hyperhomocysteinemic group (mean: 53.5 years) was older than the normohomocysteinemic group (mean: 49.9 years) and included more women. There were six women (20.68%) and three men (%9.93%) in hyperhomocysteinemic group. In the hyperhomocysteinemic group, five patients had low folic acid level, one had low vitamin B12 level, and two had low vitamin B6 level (Table-I).

**Table-I:** Distribution of hyperhomocysteinemic patients according to their age, gender, folate, vitamin B12, & vitamin B6 concentrations.

Age & gender	Homocysteine (µmol/L)	Folic acid (ng/ml)	Vitamin B12 (pmol/L)	Vitamin B6 (nmol/L)
41 Female	19.07	2.73	209.44	55.42
57 Female	17.74	5.64	679.92	122.13
65 Female	31.28	2.36	247.22	23.95
69 Female	16.03	2.43	133.33	37.78
50 Female	21.24	4.39	717.57	46.78
51 Male	15.01	4.20	782.43	104.10
43 Female	21.70	3.73	285.06	31.11
66 Male	24.70	1.97	439.02	50.60
40 Male	16.89	1.96	314.02	41.00

Low folic acid levels were observed in 18 (62%) female and 8 (25.8%) male patients. Mean age of the patients with low folic acid level (50.3 years) was slightly lower than those with normal folic acid level (51.7 years).

Although MTFR mutation analysis was not planned in this study, subsequently MTHR 677 and MTHFR 1298 mutations analysis were incidentally evaluated from the our continuing study. Mutation analysis was performed in 22 patients, of whom 17 patients had normal serum homocysteine concentration. In these 17 patients, MTHFR 1298 heterozygote mutation was found in 5 patients, MTHFR 1298 homozygote mutation was found in two patients, MTHFR 677 heterozygote mutation was found in two patients, MTHFR 677 homozygote mutation was found in two patients, compound heterozygosity for MTHFR 1298 & MTHFR 677 was found in 3 patients. There were no MTHFR mutations in three patients. In the remaining five patients who had hyperhomocysteinemia; MTHFR 1298 homozygote mutation was found in two patients, combined MTHFR 1298 heterozygote and MTHFR 677 heterozygote mutations were found in 3 patients.

## DISCUSSION

Homocystein is a nonessential amino acid containing sulfur that is formed by demethylation pathway of the methionine, which is dependent on vitamins (folate, vitamin B12 and vitamin B6) as cofactors or cosubstrates.^8^,^9^ Homocysteine is synthesized by two metabolic pathways (remethylation and transsulfuration). These pathways require vitamin B12 & folate for methionine synthesis & pyridoxal-5 phosphate for cystathionine synthesis.^10^

Homocysteine metabolic pathways involve many vitamins such as vitamin B6, vitamin B12 and folic acid. It has been thought that the main pathophysiological link among these vitamins and venous thrombosis is accumulation of homocysteine owing to decreased concentrations of these B group vitamins. However, all of these vitamins have a homocysteine-independent role related with development of venous thrombosis.^11^ Additionally, hyperhomocysteinemia inhibits the inactivation of factor Va by activated protein C and could increase the effect of factor V Leiden (FVL).^12^

Hyperhomocysteinemia may be related to genetic (cystathione β-synthase deficiency or thermolabile variant of MTHFR) or deficiencies of related B group vitamins.^13^ Severe hyperhomocysteinemia (>100 µmol/L) is most often caused by the cystathionine β-synthase deficiency.^14^ Mild or moderate hyperhomocysteinemia may result from a relative deficiency of folic acid and vitamin B12 and homozygosity for the common polymorphism in the (MTHFR) gene (677CT).^14^ None of our patients had severe hyperhomocysteinemia.

Increased homocysteinemia levels have toxic effects to the vascular structure.^13^ Therefore, hyperhomocysteinemia has been related with a wide range of disorders such as cardiovascular diseases, stroke, and venous thrombosis.^9^ In our study, low folate levels were found to be related with hyperhomocysteinemia in patients with DVT. MTHFR (C677T) polymorphism is the most frequently investigated hereditary abnormality responsible for hyperhomocysteinemia. This variation causes a decrease in the activity of the enzyme, so an increase in the homocysteine level is present.^9^ In a study conducted by Hainaut et al.^5^ prevalence of this thermo labile variant (MTHFR 677 T) was found to be similar in patients with VTE and healthy controls (13% vs 12%). Therefore, Screening of VTE patients for this thermolabile polymorphism was found unnecessary. On the other hand, Koc and Akar^10^ reported highest susceptibility to increased homocysteine levels in homozygous individuals with MTHFR 677 TT polymorphism. Nevertheless, determination of homocysteine level is more important than DNA diagnosis of MTHFR 677 mutation. In our series, 2 (9.1%) of the 22 patients who underwent genetic analysis had homozygous MTHFR 677 polymorphism.

Several clinical studies reported more prevalent increase in homocysteinemia in patients with venous thrombosis than controls.^5^ However, the association between hyperhomocysteinemia and venous thrombosis remains controversial.^17^ In Turkish healthy individuals, plasma homocysteine levels reported between 10 and 12 µmol/L.^9^

Hainaut et al.^5^ reported that 11.2% of VTE patients had hyperhomocysteinemia. According to our knowledge, there are two studies assessing the prevalence of hyperhomocysteinemia in patients with VTE from our country. Kokturk et al.^9^ reported the 43% prevalence of hyperhomocysteinemia in patients with VTE. But, Okumus et al.^16^ reported similar prevalence of hyperhomocysteinemia between patient and control groups (11.5% vs 8.9%, p>0.5). In our study, the prevalence of hyperhomocysteinemia (15%) is in agreement with previous studies.^9^

In this study, there were 26 patients (43.3%) with low folate concentration, of whom five had hyperhomocysteinemia. This study implies that hyperhomocysteinemia and low folate intake may be a risk factor for deep venous thrombosis.

Adding dietary B vitamins (folate, vitamin B6, and vitamin B12) can lower homocysteinemia concentrations in almost all patients.^18^ Nevertheless, it is not known whether homocysteine lowering therapy such as folic acid, vitamin B6 or vitamin B12 supplementation can modify thrombogenic potential of hyperhomocysteinemia in prevention of recurrent venous thrombosis.^19^

Normal folate levels ranges may vary widely in different populations and genders due to the special lifestyle factors. In this series, there were more female patients with low folate level (62%) than male patients (25.8%). Home-cooked meals are not prepared to preserve the vitamins and have a low content of meat in our urban population. Prolonged cooking of vegetable dishes may result in diminished folate content.^20^ Unlike women, men often have eaten their lunch (enough dietary meat and accurate cooking) at their working place. Refectories of working places are supervised by dietitians. Prolonged cooking of vegetables and low dietary meat content may be possible explanation for lower serum folate levels in women.

In this series, mean homocystein was significantly higher in patients over the age of 40 years than younger patients. Although, in elderly patients, hyperhomocysteinemia can also referred to impaired kidney function, low blood folate levels and increased incidence of blood vitamin B12 insufficiency associated with the aging gut, the relationship between aging and hyperhomocysteinemia is not well understood.^20^

Our data suggest that hyperhomocysteinemia, especially in women older than 40 years, may be a risk factor for DVT. Further studies are required to solve the underlying biochemical mechanisms.^21^ For the time being, folate therapy may be offered to the patients with deep venous thrombosis in Turkish population. However, further investigation in other populations would be of benefit.

Our study is limited by its small number of patients. Larger series of patients with healthy control groups might illuminate these important issues.
